# Ultraviolet (UV-C) inactivation of *Enterococcus faecium*, *Salmonella choleraesuis* and *Salmonella typhimurium* in porcine plasma

**DOI:** 10.1371/journal.pone.0175289

**Published:** 2017-04-11

**Authors:** Elena Blázquez, Carmen Rodríguez, Jesús Ródenas, Ana Pérez de Rozas, Joaquim Segalés, Joan Pujols, Javier Polo

**Affiliations:** 1 APC EUROPE, S.A., Pol. Ind. El Congost, Granollers, Spain; 2 IRTA, Centre de Recerca en Sanitat Animal (CReSA, IRTA-UAB), Campus de la Universitat Autònoma de Barcelona, Bellaterra, Barcelona, Spain; 3 Departament de Sanitat i Anatomia Animals, Universitat Autònoma de Barcelona (UAB), Bellaterra, Barcelona, Spain; 4 UAB, Centre de Recerca en Sanitat Animal (CReSA, IRTA-UAB), Campus de la Universitat Autònoma de Barcelona, Bellaterra, Barcelona, Spain; Humboldt-Universitat zu Berlin, GERMANY

## Abstract

The objective of this study was to assess the effectiveness of an ultraviolet (UV-C, 254 nm) irradiation system on reducing the load of *Salmonella typhimurium* (*S*. *typhimurium*), *Salmonella choleraesuis (S*. *choleraesuis)* resistant to streptomycin and *Enterococcus faecium* (*E*. *faecium*) inoculated in sterile porcine plasma and then subjected to different UV-C irradiation doses (750, 1500, 3000, 6000 and 9000 J/L) using a pilot plant UV-C device working under turbulent flow. Results indicated that UV-C treatment induced a viability reduction of 0.38, 1.18, 3.59, 4.72 and 5.06 log10 *S*. *typhimurium* when irradiated at 750, 1500, 3000, 6000 and 9000 J/L, respectively. The observed log10 reduction of *S*. *choleraesuis* was 1.44, 2.68, 5.55, 7.07 and 7.97 at 750, 1500, 3000, 6000 and 9000 J/L, respectively. The best-fit inactivation for *S*. *choleraesuis* was the Weibull distribution curve, while the best-fit curve for *S*. *typhimurium* was the Weibull plus tail model, indicating that around 10^2^ cfu/mL resistant *S*. *typhimurium* was detected when the liquid plasma was UV-C irradiated at doses up to 9000 J/L. Viability reduction for *E*. *faecium* was 0.44, 1.01, 3.70, 5.61 and 6.22 log10 when irradiated at 750, 1500, 3000, 6000 and 9000 J/L, respectively, with no bacterial resistance observed with UV-C doses of 6000 J/L or higher. The biphasic model was the best fit model for the inactivation curve for *E*. *faecium*. For the three microorganisms tested, about a 4 log-unit reduction was achieved when the liquid plasma was irradiated at 3000J/L. Overall results demonstrate the usefulness of the UV-C system to inactivate bacteria in liquid plasma before spray-drying. We conclude that the UV-C system can provide an additional biosafety feature that can be incorporated into the manufacturing process for spray-dried animal plasma.

## Introduction

Spray-dried plasma (SDP) is a functional protein source used in pig diets due to its beneficial effects on post-weaning performance and survival [1]. The manufacturing process for SDP involves several safety features including collection from healthy animals, pooling of inherent antibodies from multiple animals, and spray-drying the liquid at high temperature, however as technology develops, additional safety features should be investigated.

Ultraviolet (UV) treatment involves the use of shortwave electromagnetic radiation for purposes of microbial inactivation. The germicidal range is considered effective around 250 and 270 nm and a wavelength of 254 nm (UV-C) has been extensively used for disinfection of water, surfaces and food products [2,3]. Exposure to UV irradiation may produce deleterious effects on bacteria and virus survival as a result of photochemical damage to their nucleic acids. Absorption of UV promotes thymine-thymine dimers (the most common ones) and thymine-cytosine dimers which can alter the union between the double chains and inhibit DNA replication and transcription. In addition, UV irradiation causes cytosine-uracyl dimerization in RNA [4].

The efficacy of microbial reduction by UV-C treatment of liquids depends on different factors, including the microorganism used, the opaqueness of the liquid, the percentage of suspended particles in the liquid, and the microbial contamination levels at the starting point [5]. The intensity of penetration of UV-C decreases with the presence of certain amounts of soluble and insoluble solids [6,7]. For this reason, a turbulent flow during liquid processing is mandatory with the objective to avoid the laminar flow of liquid and increase the probability that all liquid content receives the same level of UV-C radiation exposure [8,9].

*Salmonella* genus is a member of *Enterobacteriaceae* family. The genus *Salmonella* can be divided into two species (*S*. *enterica* and *S*. *bongori*) and *S*. *enterica* can be further subdivided into six subspecies. *Salmonella enterica var*. *enterica serovar typhimurium* is a cause of acute foodborne zoonosis worldwide [10] and pigs are important reservoirs [11]. *Salmonella enterica var*. *enterica serovar choleraesuis* is frequently reported in North America and Asia [12,13] causing disease in pigs, with a lower prevalence reported in Europe. *Enterococcus faecium* (*E*. *faecium*) NRRL B-2354 has been used as a model organism in thermal validation studies and is considered a suitable surrogate for foodborne pathogens to validate thermal processes used for dairy products, almonds, liquid foods and meat [14,15].

The objective of this study was to assess bacterial inactivation efficiency of UV-C irradiation of liquid porcine plasma using a pilot plant system designed for irradiation of opaque liquids subjected to a turbid flow. The inactivation efficacy of the UV-C treatments was determined for *S*. *typhimurium* and *S*. *choleraesuis*, as well as *E*. *faecium*.

## Material and methods

### Bacterial strains and culture conditions

*S*. *typhimurium* (*ref UNI-UAB 46450)* and *S*. *choleraesuis (ref UMI-UAB 46429)* strains were provided by the UMI-UAB (Veterinary School, Infectious Diseases Unit, *Universitat Autònoma de Barcelona*, Spain). They were cultured in several passes in tryptic soy agar (TSA) (Sigma-Aldrich) with increased amount of streptomycin from 0 to 500 μg/mL. Only colonies resistant to 500 μg streptomycin/mL of culture medium were used for the study. The antibiograms and the MIC showed a similar resistant profile for both *Salmonella* spp. They were resistant to Ampicillin, Ciprofloxacin, Nalidixic acid, Gentamicin, Streptomycin, Tetracycline, Colistin, Sulfamethoxazole, Trimethoprim, Chloramphenicol, Kanamycin, Ceftazidime, Trimetoprim+sulfamethoxazole, Neomycin, Rifampicin, Tiamulin and Tylosin.

*Enterococcus faecium* (strain NRRL B-2354, ATCC 8459) was grown in brain-heart infusion agar (BHIA) (Sigma-Aldrich).

### Inoculum preparation

*Salmonella* spp. inocula for both strains were prepared in TSA media containing 500 μg steptomycin/mL. After 24 hours of growth at 37°C, bacteria were harvested by a Kolle handle and resuspended in 10 mL PBS. The inoculum for *E*. *faecium* was prepared after growth in BHIA for 24 h at 37°C.

Liquid fresh plasma from industrial abattoirs may contain different microorganisms. For that reason, it was decided to sterilize 2.5 kg of spray-dried porcine plasma (lAP820P, APC-Europe S.A., Granollers, Spain) by gamma-cobalt-60 irradiation at 10 KGray (Aragogamma S.A, Les Franqueses del Vallés, Barcelona, Spain) to eliminate any potential bacteria. The γ-irradiated SDP was diluted 1:11 in water (2.5 kg SDP + 25.0 kg of water) to obtain 27.5 kg of liquid plasma at 9.66, 9.50 and 9.11% solids for *S*. *choleraesuis*, *S*. *typhimurium* and *E*. *faecium*, respectively. An inoculum of each individual bacterium containing 10^9^ cfu/mL was prepared and used to infect 24 L aliquots of plasma to achieve a minimum final titer of approximately 10^6^ cfu/mL. All bacterial handlings were done in a sterile laminar flow cabin to protect the staff and microbiological cultures.

### UV-C irradiation

After mixing the bacteria inoculum with the 24 aliquot of plasma, the total volume was divided into three 8 L sub-aliquots sub. At time zero, a positive control 15 mL sample was collected 5 min. after bacteria were mixed with the plasma. Samples of each sub-aliquot were consecutively irradiated at 750, 1500, 3000, 6000 and 9000 J/L. During the UV-C treatment, sequential 15 mL samples were taken at each time-dose of irradiation. As a negative control, a 15 mL sample of liquid plasma was obtained before bacteria inoculation.

After UV-C irradiation, 1 mL samples were ten-fold diluted in peptone water and 0.1 mL of the dilatant was inoculated onto 25 mL TSA plates containing streptomycin (500μg/mL) to count *S*. *typhimurium*, or *S*. *choleraesuis* and 0.1mL of the dilatants was inoculated onto 25 mL BHIA plates for count of *E*. *faecium* colonies that survived the different UV-C irradiation doses.

Count results were expressed as a Log 10 CFU/mL, and survival curves were plotted as Log10/mL as function of UV-C dose (J/L).

### Settings of pilot scale UV-C system

**Fig 1:**
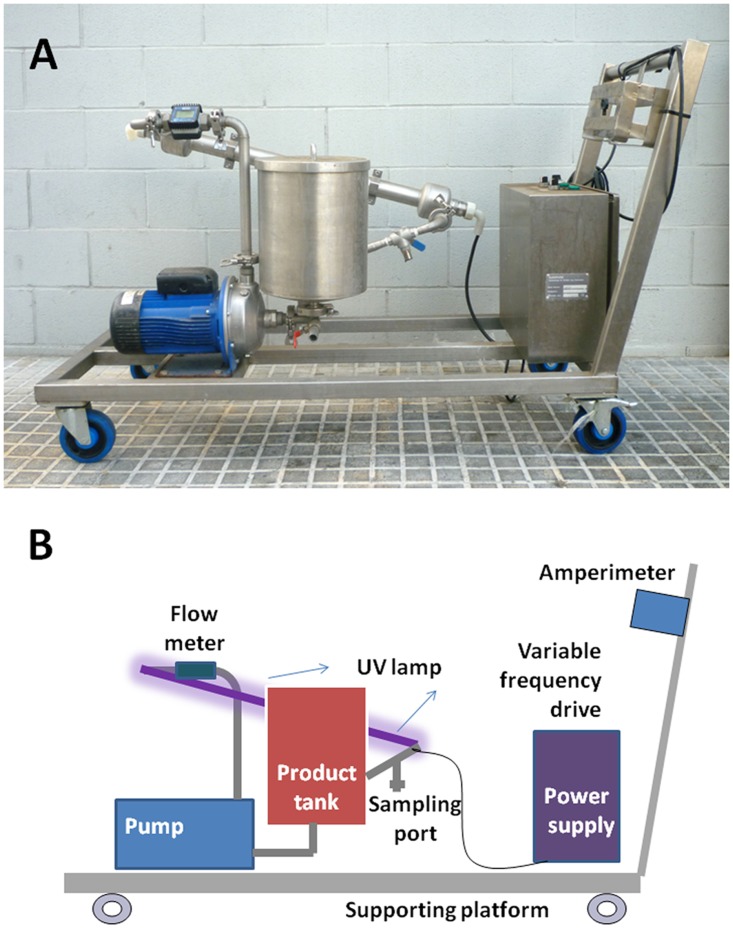
A. Picture of the UV system SP1 used in the experiments. B. Diagram of the different elements of the system.

The UV-C reactor system (SP1, Fig 1) was designed and manufactured by Sure Pure Operation AG (Zug, Switzerland), and consists of a closed system with one low pressure mercury UV lamp (30 UVC Watts, 254 nm) surrounded by a quartz crystal which avoids contact with the product. The plasma flows through a steel tube containing a vortex (internal striped spiral tube), which generates a turbulent flow. The liquid flows between the corrugated spiral tube and the quartz sleeve. The tangential inlet of the reactor creates a high velocity and turbulence in the inlet chamber and brings the liquid into contact with the UV-C radiation. The liquid is pumped from the inlet chamber into the reactor, through the gap between the quartz sleeve and the corrugated spiral tubing, at a minimum flow rate of 3800 L/h with a Reynolds value in excess of 7500, indicating turbulent flow. Plasma was recirculated by the pump from the tank to the UV-C lamp and recirculated many times through this circuit to achieve the required UV-C dose versus time. Liquid flow was controlled by a flow meter. Flow rate was adjusted to 4000 L/h. The time spent by the liquid (8L total volume) to pass through the system once was 7.2 s, delivering 22.95 J/L or 23.40 mJ/cm^2^ per cycle.

At the start of the process, the flow rate was stabilized for 5 min water recirculation. Water was replaced by plasma and then the treatment process was initiated. First, with the UV-C lamp turned off, a positive control (time 0) sample was collected allowing the product to recirculate throughout the SP1 system at 4000 L/h during 5 minutes. After this step, the UV-C lamp was switched on and irradiation was started. Samples were collected at defined times into sterile containers for microbiological analysis. Samples were collected at different UV-C doses (0, 750, 1500, 3000, 6000 and 9000 J/L) corresponding to different intervals of irradiation time (0, 4’31”, 7’49”, 15’35”, 31’05” and 46’28”).

### Modeling of inactivation

Bacterial inactivation due to thermal and non-thermal processes can display one of eight possible curve shapes. Log linear inactivation modeling fails to accurately assess the majority of the survival curves [16].

In order to analyze the inactivation curves and to assess if bacteria inactivation was linear or non-linear, the GInaFiT software was used to obtain non-linear survival curves [16] and to test log-linear plus tail [17], Weibull [18], Weibull plus tail [19] and biphasic [20] models.

The log-linear plus tail model uses the Eq (1): [16]

$$logN=\text{log}\left(\left(10^{\text{log}N0}-10^{logN_{res}}\right)\right)-e^{\left(\;k_{max}\;d\right)}+10^{logN_{res}}$$ (1)

Where $k_{max}$ is the inactivation rate of the log linear part of the curve.

The biphasic model uses the Eq (2): [20]

$$log10(N)=log10(N0)+log10\;(f*e^{-k_{max}{}_{1}t}+(1-f)*e^{-k_{max}{}_{2}t})$$ (2)

The Weibull model uses the Eq (3): [18]

$$log10\left(N\right)=log10\left(N\left(0\right)\right)-(\frac{t}{\delta }){\;}^{p}$$ (3)

Where δ is a scale parameter denoted as the time for the first decimal reduction, and p is the shape parameter that describes concavity or convexity of the curve. If p>1 the curve shows convexity and if p<1 the curve is concave.

And the Weibull model plus tail follows the Eq (4): [19]

$$log10\left(N\right)=log10\;\left(\left(10^{\text{log}N0}-10^{logN_{res}}\right)\right)\;*\;10^{\left(-\frac{t}{\delta }\right){\;}^{p}}+10^{logN_{res}}$$ (4)

$Where\;N_{res}$ is the number of resistant bacterial subpopulation.

### Statistical analysis

Data were expressed by means of Log10 values and standard deviations of three independent experimental batches.

Mean, standard deviations, ANOVA and F-Test for comparisons were calculated with Excel 2007 (Microsoft Office) to determine significant differences between doses. Tukey test was calculated with Statgraphics Centurion XV version 15.2.14 (StatPoint Technologies Inc, Warrenton, Virginia) to determine significant differences between treatments for both Salmonella and *E*. *faecium*. Differences at p<0.05 were considered significant.

Mean square error (MSE), goodness of fit in terms of root mean square error (RMSE), correlation coefficient (R^2^) and adjusted correlation coefficient (adj-R^2^) values were calculated with GInaFiT software [16]. To choose the inactivation model with the best fit, the model with the smallest RMSE was chosen [16].

## Results

There were significant differences (p<0.05) between treatments for the three tested bacteria, except for the first treatment on *S*. *typhimurium* (750 J/L), which was not significantly different from time 0.

**Fig 2:**
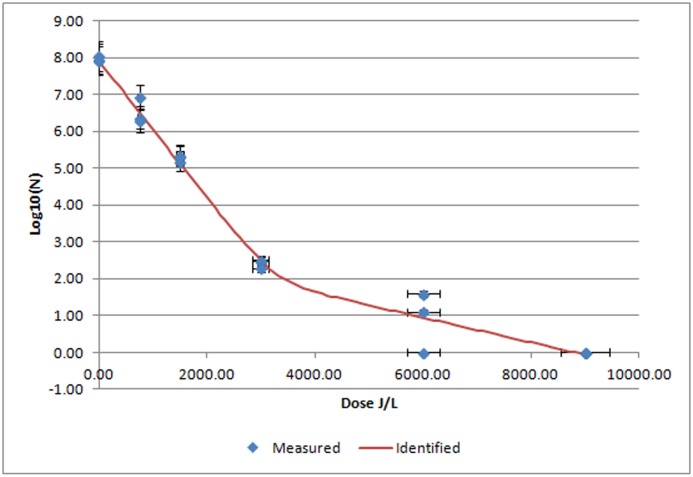
Mean *S*. *choleraesuis* log 10/mL values after UV-C irradiation of porcine plasma at different UV irradiation doses. Blue diamonds indicated measured results of *S*. *choleraesuis* at different UV-C irradiation doses expressed as mean ± SEM (n = 3 replicates). Red line is the identified curve according to the biphasic inactivation curve model.

Plasma inoculated with *S*. *choleraesuis* had an initial count of 7.97 log10/mL and when UV-C treated showed a curve with a regression coefficient of R^2^ = 0.9867 (Table 1). *S*. *choleraesuis* displayed a robust and replicable reduction rate of 5.5 log10 between 0 and 3000J/L of UV-C. However, a complete inactivation of *S*. *choleraesuis* (7.97 log) was achieved at 9000 J/L (Fig 2)

**Table 1:** Statistical parameters of three models for inactivation of *S*. *choleraesuis*, *S*. *typhimurium* and *E*. *faecium*.

	*S*. *choleraesuis*		*S*. *typhimurium*		*E*. *faecium*	
	Biphasic	Weibull	Biphasic	Weibull plus tail	Biphasic	Weibull
**MSE**^1^	0.1495	0.5116	0.1367	0.0588	0.2577	0.5420
**RMSE**^2^	**0.3867**	0.7152	0.3698	**0.2425**	**0.5076**	0.7362
**R-Square**	0.9867	0.9511	0.9698	0.9870	0.9684	0.9288
**R-Square adjusted**	0.9838	0.9446	0.9622	0.9837	0.9617	0.9193
**4D reduction is reached at (J/L)**^3^	**2301**	2125	3364	**3186**	**3364**	3984

^1^MSE: Mean sum of squared error.

^2^RMSE: Root mean sum of squared error.

^3^4D reduction: UV irradiation in J/L at which achieved 4 Log reduction.

Data for *S*. *choleraesuis* fitted slightly better with the biphasic model having the lowest RMSE (Table 1)

**Fig 3:**
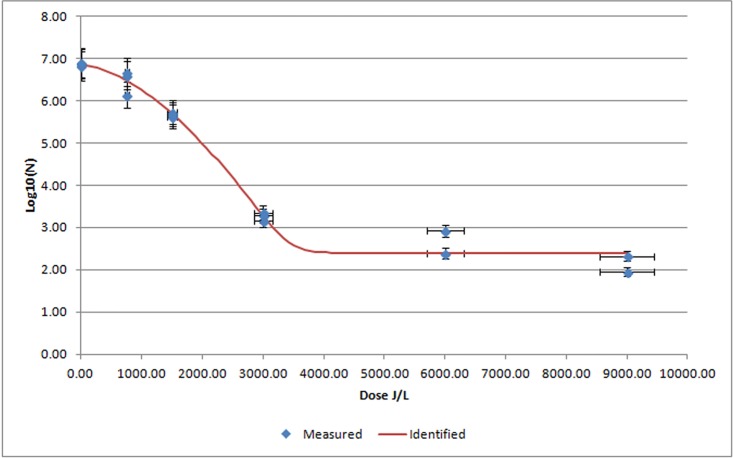
Mean *S*. *typhimurium* log 10/mL values after UV-C irradiation of porcine plasma at different UV irradiation doses. Blue diamonds indicate measured results of *S*. *typhimurium* at different UV-C irradiation doses expressed as mean ± SEM (n = 3 replicates). Red line is the identified curve according to the Weibull plus tail inactivation curve model.

Plasma inoculated with *S*. *typhimurium* had an initial count of 6.85 ± 0.04 log10/mL and, after UV-C treatment, the decrease in bacterial counts showed a curve adjusted with the Weibull plus tail model, with a regression coefficient of R^2^ = 0.9870 (Table 1). *S*. *typhimurium* displayed a robust and replicable inactivation of 3.59 log10 between 0 and 3000 J/L (Fig 3). A residual of around 2 log of *S*. *typhimurium* counts was observed after irradiation at a dose of 9000 J/L.

**Fig 4:**
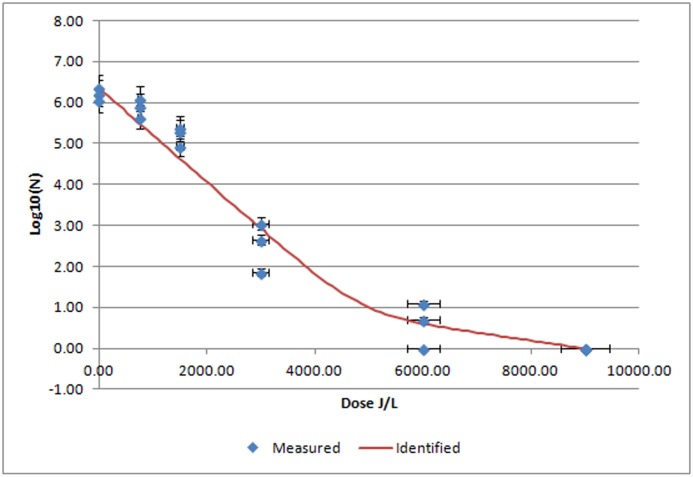
Mean *E*. *faecium* log 10/mL values after UV-C irradiation of porcine plasma at different UV irradiation doses. Blue diamonds indicated measured results of *E*. *faecium* at different UV-C irradiation doses expressed as mean ± SEM (n = 3 replicates). Red line is the identified curve according to the biphasic inactivation curve model.

*E*. *faecium* was inoculated at 6.22 ± 0.13 log10/mL in liquid plasma and after UV-C treated, a biphasic growth curve with a regression coefficient of R^2^ = 0.9684 was observed (Table 1, Fig 4). At UV-C irradiation dose of 9000 J/L there was a total lack of detectable bacterial growth.

## Discussion

Plasma is obtained from blood of farm animals in slaughterhouses and constitutes the raw material to obtain a dehydrated product used as a nutritive feed additive. SDP is a common ingredient in post-weaning pig diets due to its beneficial effects on performance and survival. SDP of porcine origin (SDPP) is considered an inherently safe protein for use in animal feed. In fact, during the manufacturing of spray-dried blood products, there are several safety features including collection of blood only from healthy animals, pooling of inherent neutralizing antibodies in plasma from multiple animals, and spray-drying at high temperature. Spray-drying has been shown effective to inactivate different swine bacteria such as *E*. *coli K88* and *K99* [21] and viruses, such as *Porcine reproductive and respiratory syndrome virus*, *Pseudorabies virus* [22], *Porcine epidemic diarrhea virus* [23,24] and *Swine vesicular disease virus* [25]. In addition, pigs fed a diet containing commercial SDPP with genome for *Porcine parvovirus* (PPV) and/or *Porcine circovirus type 2* (two of the most thermal and solvent resistant known viruses) were not infected [22, 26–28], suggesting commercial manufacturing processes for SDPP were sufficient for inactivation of these two high heat resistant swine viruses.

However, as microbes evolve and new pathogens are discovered, it is prudent to evaluate the introduction of additional safety features that should further enhance the robustness of the manufacturing process for SDP. The efficacy of UV-C irradiation is limited by the penetration capacity of the light into a liquid based upon the solids content of the liquid [29]. Therefore, the application of a UV-C system to turbid liquids like animal plasma require a special design to assure that the liquid has a turbulent flow regime to enable UV-C light to penetrate the liquid to be irradiated. The Sure Pure patented system (EP-1255444B1) creates a turbulent flow (Reynolds number > 2800) to allow the surface area of the liquid to be exposed to UV-C through a very narrow gap in the irradiation chamber. This system has been proven to be able to process large volumes of raw liquids like fruit juices, wine [8,30] and milk [31]. Therefore, it is a well-developed technology suitable to be applied to the animal blood product business. In addition, UV-C irradiation is widely recognized as a safe technology for inactivation of microbes in food or feed products without causing negative effects on the nutritional or physical qualities of the treated material [32,33].

In a previous study using a similar pilot plant Sure Pure system (SP1) [34], UV irradiation of liquid plasma at doses higher than 2295 J/L inactivated more than 10^5.2^ TCID_50_/mL of PPV inoculated in the liquid plasma. Also in the same publication UV-C irradiation of liquid plasma prior to spray-drying did not negatively affect the productive parameters of post-weaned pigs fed a diet containing UV-C treated SDPP. Although UV-C irradiation was effective for inactivation of PPV, a model for heat resistant viruses, it was also necessary to investigate the effect of UV-C irradiation on the survival of different bacteria of interest such as *S*. *typhimurium*, *S*. *choleraesuis* and *E*. *faecium* when inoculated in liquid plasma. Bacteria have more mechanisms of genome reparation than viruses and it can be argued that the effect of UV-C irradiation may be less efficient in bacteria compared with simpler microorganisms as viruses. In addition, it was necessary to know if bacteria that are able to develop antibiotic resistance may be more difficult to eliminate when UV-C irradiation is applied. In the present experiments, a reduction of almost 4 log for each bacterium was determined when irradiation with UV-C doses around 3000J/L were used. According to calculations, a four decimal (4D) reduction was reached at 2301 J/L for *S*. *choleraesuis*, 3186 J/L for *S*. *typhimurium* and 3364 J/L for *E*. *faecium* when using the best-fit inactivation model curve.

*E*. *faecium* and *S*. *choleraesuis* had total inactivation by UV-C at 9000 J/ L with a biphasic curve in both cases. However, *S*. *typhimurium* displayed an inactivation resistance with a tail effect. The tail survival effect of *S*. *typhimurium* may be related to a bacteria resistant subpopulation. *S*.*typhimurium* showed a N*res* parameter (Number of resistant bacterial subpopulation in a Weibull plus tail model of 2.40 log ±0.12. These results agree with data obtained by Luksiene et al. [35], who found a similar distribution of inactivation results working with pulsed UV-light. Usually, microorganism inactivation by UV-C light follows a first order kinetics in liquids, but some agents can exhibit a sigmoidal shape with shoulder or tail curves [36]. In the present work, the GINAFiT software [16] was used to test the best-fit model for the obtained data, using the goodness of fit in terms of RMSE to select the model for each individual bacterial inactivation curve. Both *Salmonella* spp. strains used in this study were resistant to streptomycin. Therefore, both strains contained genes involved in antibiotic resistance, but UV-C inactivation curves for both strains were different; a higher UV-C resistance was found for *S*. *typhimurium* compared to *S*. *choleraesuis*. These results may suggest that the genes involved in antibiotic resistance may not preclude the resistance of these bacteria to UV irradiation and different gene resistance development or resistance mechanism may be involved.

*E*. *faecium* is a food-borne surrogate bacterium with no antibiotic resistance genes that is commonly used to compare its inactivation kinetics with those obtained with pathogenic bacteria in industrial manufacturing processes. In this study, the inactivation curve for *E*. *faecium* was similar to the inactivation curve for *S*. *choleraesuis*, although each bacterium had different best-fit inactivation curves. Therefore, it is suggested that *E*. *faecium* can be used as food surrogate for this bacteria in UV-C studies for animal plasma conducted at a commercial level. In contrast, the inactivation behavior of *E*. *faecium* was significantly different to the curve observed for *S*. *typhimurium*, suggesting that this food probiotic bacterium is not a good surrogate for *S*. *typhimurium*.

In conclusion, these results provide evidence of the 4D inactivation of studied microorganisms with affordable levels of UV-C irradiation. In addition, results suggested that UV-C technology can be used as an additional biosafety feature to minimize risk of biohazards that may be present in biological products like liquid plasma.

## Supporting information

S1 Table *Enterococcus faecium* log 10 reduction for each triplicate at each time/dose. Dose was calculated as a UV-fluence received per unit of time. These data were used for GInaFiT analysis.
(DOCX)

S2 Table *Enterococcus faecium* log 10 reduction in terms of mean, and the step log reduction and total log reduction at each time/dose. (DOCX)

S3 Table *Salmonella choleraesuis* log 10 reduction at each time/dose. Dose was calculated as a UV-fluence received per unit of time.
(DOCX)

S4 Table *Salmonella choleraesuis* log 10 reduction in terms of mean, and the step log reduction and total log reduction at each time/dose. (DOCX)

S5 Table *Salmonella typhimurium* log 10 reduction at each time/dose. Dose was calculated as a UV-fluence received per unit of time.
(DOCX)

S6 Table *Salmonella typhimurium* log 10 reduction in terms of mean, and the step log reduction and total log reduction at each time/dose. (DOCX)
