# Supplementary material for: Ultraviolet (UV-C) inactivation of Enterococcus faecium, Salmonella choleraesuis and Salmonella typhimurium in porcine plasma
Source: PLoS One. 2017 Apr 11;12(4):e0175289. doi: 10.1371/journal.pone.0175289 (PMC5388490; doi:10.1371/journal.pone.0175289)
Supplement: S1 Table — Dose was calculated as a UV-fluence received per unit of time. These data were used for GInaFiT analysis. (DOCX) [file pone.0175289.s001.docx]

| ***Enterococcus faecium*** | | |
| --- | --- | --- |
| **DOSE (J/L)** | **TIME (min)** | **Log10/mL** |
| 0 | 0 | 6.35590002 |
| 0 | 0 | 6.23394956 |
| 0 | 0 | 6.06948636 |
| 750 | 4.31 | 6.08462681 |
| 750 | 4.31 | 5.9101646 |
| 750 | 4.31 | 5.65331448 |
| 1500 | 7.49 | 5.31822656 |
| 1500 | 7.49 | 5.39039439 |
| 1500 | 7.49 | 4.93215139 |
| 3000 | 15.35 | 3.038632 |
| 3000 | 15.35 | 2.65326921 |
| 3000 | 15.35 | 1.868867 |
| 6000 | 31.05 | 1.1 |
| 6000 | 31.05 | 0.7155665 |
| 6000 | 31.05 | 0 |
| 9000 | 46.28 | 0 |
| 9000 | 46.28 | 0 |
| 9000 | 46.28 | 0 |

**S1 Table 1. *Enterococcus faecium* log 10 reduction for each triplicate at each time/dose**. Dose was calculated as a UV-fluence received per unit of time. These data were used for GInaFiT analysis.
