# Supplementary material for: Ultraviolet (UV-C) inactivation of Enterococcus faecium, Salmonella choleraesuis and Salmonella typhimurium in porcine plasma
Source: PLoS One. 2017 Apr 11;12(4):e0175289. doi: 10.1371/journal.pone.0175289 (PMC5388490; doi:10.1371/journal.pone.0175289)
Supplement: S2 Table — (DOCX) [file pone.0175289.s002.docx]

| ***Enterococcus faecium*** | | | | | |
| --- | --- | --- | --- | --- | --- |
| **DOSE J/L** | **TIME (s)** | **MEAN** | **SD** | **Step log reduction** | **Log reduction from control** |
| 0 | 0 | 6.22 | 0.13 | 0.00 | 0.00 |
| 750 | 4.31 | 5.78 | 0.15 | 0.44 | 0.44 |
| 1500 | 7.49 | 5.21 | 0.22 | 1.01 | 1.01 |
| 3000 | 15.35 | 2.52 | 0.53 | 2.69 | 3.70 |
| 6000 | 31.05 | 0.61 | 0.67 | 1.92 | 5.61 |
| 9000 | 46.28 | 0.00 | 0.00 | 0.61 | 6.22 |
| Acumulated reduction 6.22 | | | | | |

**S2 Table 2. *Enterococcus faecium* log 10 reduction in terms of mean, and the step log reduction and total log reduction at each time/dose.**
