# Supplementary material for: Ultraviolet (UV-C) inactivation of Enterococcus faecium, Salmonella choleraesuis and Salmonella typhimurium in porcine plasma
Source: PLoS One. 2017 Apr 11;12(4):e0175289. doi: 10.1371/journal.pone.0175289 (PMC5388490; doi:10.1371/journal.pone.0175289)
Supplement: S3 Table — Dose was calculated as a UV-fluence received per unit of time. (DOCX) [file pone.0175289.s003.docx]

| ***Salmonella choleraesuis*** | | |
| --- | --- | --- |
| **DOSE (J/L)** | **TIME (min)** | **Log10/mL** |
| 0 | 0 | 7.90761861 |
| 0 | 0 | 8.03468932 |
| 0 | 0 | 7.96181879 |
| 750 | 4.31 | 6.91881182 |
| 750 | 4.31 | 6.37422654 |
| 750 | 4.31 | 6.29198016 |
| 1500 | 7.49 | 5.18680732 |
| 1500 | 7.49 | 5.34947579 |
| 1500 | 7.49 | 5.31529444 |
| 3000 | 15.35 | 2.4740708 |
| 3000 | 15.35 | 2.50596638 |
| 3000 | 15.35 | 2.28709937 |
| 6000 | 31.05 | 1.1 |
| 6000 | 31.05 | 1.59669949 |
| 6000 | 31.05 | 0 |
| 9000 | 46.28 | 0 |
| 9000 | 46.28 | 0 |
| 9000 | 46.28 | 0 |

**S3 Table 3. *Salmonella choleraesuis* log 10 reduction at each time/dose.** Dose was calculated as a UV-fluence received per unit of time.
