# Supplementary material for: Ultraviolet (UV-C) inactivation of Enterococcus faecium, Salmonella choleraesuis and Salmonella typhimurium in porcine plasma
Source: PLoS One. 2017 Apr 11;12(4):e0175289. doi: 10.1371/journal.pone.0175289 (PMC5388490; doi:10.1371/journal.pone.0175289)
Supplement: S4 Table — (DOCX) [file pone.0175289.s004.docx]

| ***Salmonella choleraesuis*** | | | | | |
| --- | --- | --- | --- | --- | --- |
| **DOSE (J/L)** | **TIME (min)** | **MEAN** | **SD** | **Step log reduction** | **Log reduction from control** |
| 0 | 0 | 7.97 | 0.05 | 0 | 0 |
| 750 | 4.31 | 6.53 | 0.3 | 1.44 | 1.44 |
| 1500 | 7.49 | 5.28 | 0.08 | 1.25 | 2.69 |
| 3000 | 15.35 | 2.42 | 0.12 | 2.86 | 5.55 |
| 6000 | 31.05 | 0.89 | 0.74 | 1.53 | 7.08 |
| 9000 | 46.28 | 0 | 0 | 0.89 | 7.97 |
| Acumulated reduction 7.97 | | | | | |

**S4 Table 4. *Salmonella choleraesuis* log 10 reduction in terms of mean, and the step log reduction and total log reduction at each time/dose.**
