# Supplementary material for: Ultraviolet (UV-C) inactivation of Enterococcus faecium, Salmonella choleraesuis and Salmonella typhimurium in porcine plasma
Source: PLoS One. 2017 Apr 11;12(4):e0175289. doi: 10.1371/journal.pone.0175289 (PMC5388490; doi:10.1371/journal.pone.0175289)
Supplement: S5 Table — Dose was calculated as a UV-fluence received per unit of time. (DOCX) [file pone.0175289.s005.docx]

| ***Salmonella typhimurium*** | | |
| --- | --- | --- |
| **DOSE (J/L)** | **TIME (min)** | **Log10/mL** |
| 0 | 0 | 6.8197444 |
| 0 | 0 | 6.87249596 |
| 0 | 0 | 6.8886962 |
| 750 | 4.31 | 6.59480846 |
| 750 | 4.31 | 6.68249365 |
| 750 | 4.31 | 6.13543482 |
| 1500 | 7.49 | 5.61670318 |
| 1500 | 7.49 | 5.72294989 |
| 1500 | 7.49 | 5.68235182 |
| 3000 | 15.35 | 3.35406365 |
| 3000 | 15.35 | 3.2833837 |
| 3000 | 15.35 | 3.15576199 |
| 6000 | 31.05 | 2.92378369 |
| 6000 | 31.05 | 2.38941575 |
| 9000 | 46.28 | 1.94923742 |
| 9000 | 46.28 | 2.3239201 |

**S5 Table 5. *Salmonella typhimurium* log 10 reduction at each time/dose.** Dose was calculated as a UV-fluence received per unit of time.
