# Supplementary material for: Ultraviolet (UV-C) inactivation of Enterococcus faecium, Salmonella choleraesuis and Salmonella typhimurium in porcine plasma
Source: PLoS One. 2017 Apr 11;12(4):e0175289. doi: 10.1371/journal.pone.0175289 (PMC5388490; doi:10.1371/journal.pone.0175289)
Supplement: S6 Table — (DOCX) [file pone.0175289.s006.docx]

| ***Salmonella typhimurium*** | | | | | |
| --- | --- | --- | --- | --- | --- |
| **DOSE J/L** | **TIME (s)** | **MEAN** | **SD** | **Step log reduction** | **Log reduction from control** |
| 0 | 0 | 6.85 | 0.04 | 0.00 | 0.00 |
| 750 | 4.31 | 6.47 | 0.31 | 0.38 | 0.38 |
| 1500 | 7.49 | 5.67 | 0.09 | 0.80 | 1.18 |
| 3000 | 15.35 | 3.26 | 0.10 | 2.41 | 3.59 |
| 6000 | 31.05 | 2.14 | 0.84 | 1.13 | 4.72 |
| 9000 | 46.28 | 1.79 | 0.56 | 0.35 | 5.06 |
| Acumulated reduction 5.06 | | | | | |

**S6 Table 6. *Salmonella typhimurium* log 10 reduction in terms of mean, and the step log reduction and total log reduction at each time/dose.**
